# Bifurcation Analysis on the Periodic Response of a Comb Drive MEMS Resonator

**DOI:** 10.3390/mi13020148

**Published:** 2022-01-19

**Authors:** Huabiao Zhang, Lijuan Zhang, Xinye Li, Dongai Wang, Tingting Liu

**Affiliations:** 1 School of Mechanical Engineering, Tianjin University of Commerce, Tianjin 300134, China; wangda@tjcu.edu.cn; 2 School of Automobile and Transportation, Tianjin University of Technology and Education, Tianjin 300222, China; 2020050004@tute.edu.cn; 3 School of Mechanical Engineering, Hebei University of Technology, Tianjin 300401, China; 1994109@hebut.edu.cn; 4 Tianjin Jinyongming Science and Technology Development Company Limited, Tianjin 300393, China; liutt@163.com

**Keywords:** comb drive resonator, bifurcation, periodic response, the method of averaging, the singularity theory

## Abstract

In this paper, we investigate the bifurcation characteristics of a comb drive MEMS resonator. The method of averaging and the residue theorem are used to get a more accurate analytical solution for the periodic response. Then, the singularity theory is employed to give the transition sets on the DC-AC voltage plane and the lateral separation-quality factor plane, which divide the planes into 9 persist regions. The corresponding bifurcation diagrams are present to discuss the jump phenomena of the periodic response, and the influences of the parameters on the amplitude-frequency response are studied. We also attempt to analyze the feasibility for the resonators working in the nonlinear regions and give the available frequency range and the available maximum amplitude of the nonlinear response. With the increase of the DC voltage, the amplitude-frequency curves change from hardening to softening, and the lateral separation has the opposite effect. The amplitude-frequency curves increase along the backbone curves with the AC voltage and quality factor. The response curves of softening or hardening characteristics have enough available frequency range and large available amplitudes, which may be more appropriate for the operation of the resonator than those of the mixture characteristics.

## 1. Introduction

Due to its advantages of suppressing nonlinearities and increasing actuation force, comb drive MEMS resonator found many applications, such as micromirrors [1], gyroscopes [2], accelerometers [3], energy harvesters [4], etc. However, the nonlinearity caused by elastic deformation and electrostatic force of the resonator may result in particular response characteristics such as hardening/ softening behaviors [5], pull-in [6], chaos [7], etc., and consequently affect the performance of the resonator. Therefore, the nonlinear dynamics of comb drive MEMS resonator attracted significant attention from many researchers.

Chang et al. [8] investigated the performance of a MEMS resonator concerning resonant frequency and quality factor under various ambient pressures, AC drive voltages, bias potentials and temperatures. Ramanan et al. [9] conducted a series of experiments with different driving voltages and ambient pressures, and discussed the feasibility of resonators utilizing the nonlinear operating region. Guo et al. [10] analyzed the effect of adhesion and crack on the dynamics of a micro comb resonator by finite element method. They found adhesion fault makes the resonance frequency higher and sensitivity reduction, while crack fault debases the resonance frequency and amplitude. Truong et al. [6] investigated the lateral pull-in effect of a comb drive transducer. The analytical critical pull-in voltage considering only the translational stiffness or the rotational stiffness of the mechanical spring and the numerical critical voltage in a general case was determined. Mukherjee et al. [11] modeled the asymmetric comb drive microstructures as an electrostatically actuated double cantilever beam to obtain different performance parameters like pull-in voltage, frequency response, etc., and discussed the effects of various critical factors on the dynamic pull-in characteristics. Mukherjee et al. [7] considered the chaotic behaviors and the control strategies of a comb drive micro-accelerometer.

To suppress the nonlinearity and improve the performance of the resonator, Shmulevich et al. [12] gave a new dynamically balanced folded beam suspension to make the response linear. Chen et al. [13] present the reduction of mechanical nonlinearity of a resonator controllably utilizing the inside tuning capacitor by established theoretical models and validated experimental results. Based on adding a new electrode on the folded beams, Khirallah [14] applied the DC or AC voltages on the electrode for parametric excitation and amplification to suppress nonlinearity and achieve a high amplitude of oscillation. Taherian et al. [15] proposed a new resonator with nonuniform varied finger lengths, derived the expressions of the electrostatic driving force, support stiffness and resonant frequency, and found the structure can increase the resonant frequency range of the resonator. Zhang et al. [16] investigated the nonlinear dynamics of a resonator with time-delayed control, and pointed out that the positive gain makes the system unstable and the negative gain leads to the disappearance of jumping phenomena. Ghasemi et al. [4] used a combination of mechanically softening and hardening springs to achieve a high tuning range of resonant frequency for energy harvesting applications. Nashat et al. [17] demonstrated that a large displacement can be obtained by reducing the pull-in voltage and increasing the overlaps. Kozinsky et al. [18] present an experimental observation of electrostatic tuning of the onset of nonlinearity of a nanomechanical resonator, and demonstrated tuning the resonant frequency of the resonator both upward and downward. Nichol et al. [19] described the use of nonlinear feedback to tune the cubic nonlinearity of a silicon nanowire resonator, and showed nonlinear feedback can increase or decrease the nonlinearity, change its sign, or even make it zero. Zhang et al. [20] reported the thermal tuning of the mechanical nonlinearity in a MEMS beam resonator and observed a significant reduction in the mechanical nonlinearity near the buckling point as electrical heat was applied to the MEMS beam. Huang et al. [21] designed a micromechanical resonator with nonmonotonic dependence of the eigenfrequency on energy, and found that the system regains certain characteristics of a linear resonator with large amplitudes near the extremum where the dispersion of the eigenfrequency is zero.

The periodic response relates directly to the performance of the resonator, which is also the focus of researchers. Zhang et al. [22] investigated the nonlinear responses and dynamics of an electrostatically actuated MEMS resonant sensor under two-frequency parametric and external excitations, and examined the effects of dynamic parameters on the frequency response, resonant frequency and peak amplitude. Kacem et al. [23] reported the experimental observation of the mixed behavior and demonstrated both analytically and experimentally tuning the bifurcation topology of this behavior via an electrostatic mechanism. Khan et al. [24,25] provided several analytical methods for studying the periodic response of nonlinear oscillator equations and applied these methods to the study of an electromechanical resonator. These new approaches were proved to be very rapid, effective, and accurate by comparing their solutions with the published results. Elshurafa et al. [5] studied analytically and numerically the spring softening and hardening phenomena of a resonator considering both the transverse and longitudinal capacitance of the combs. According to the variation of softening/hardening characteristics of the resonator, Han et al. [26] classified the system response on the plane of the extremely amplitude and DC voltage. Zhong et al. [27] investigated the effects of the inclination of the fingers and edge effect on the capacitance, driving electrostatic force, and electrostatic spring constant of a resonator considering nonlinear air damping. The results demonstrated that the inclination causes the resonance frequency to increase and the electrostatic spring to harden under applied DC voltage. Ma et al. [28] researched the static and dynamic behavior of a MEMS comb resonator by using the electrostatic force with edge effect fitted by the least square method.

The existing studies are mainly based on experiments, numerical simulations and finite element analysis. The analytical investigation is helpful to get the internal law of the resonator, which is significant for the design and operation of the resonator. The electrostatic force of the comb drive resonator is generally fractional. Of most analytical investigations, the electrostatic force is fitted to a polynomial of the displacement, which inevitably leads to solution errors. In references [5,26,27], the dynamic equation with fractional nonlinearity is solved directly to give a more accurate solution. However, the harmonic function term including the ratio of the AC voltage to DC voltage is ignored in their calculations, so that the conclusions obtained may not reflect the influence of the AC voltage on the response very well. Moreover, many researchers focus on the bifurcation behaviors of the resonator, and demonstrate the amount of softening, hardening and mixed response. Actually, it is also valuable to classify the bifurcation behaviors and find the boundary of different kinds of responses on the parameter plane.

In this paper, we proposed a study on the bifurcation characteristics of a comb drive MEMS resonator. The method of averaging and the residue theorem are used to obtain a more accurate analytical solution for the periodic response. The singularity theory is employed to analyze the bifurcation characteristics and give the transition sets on the parameter planes, which may provide some theoretical basis for the selection of the structural parameters and voltages. We also consider the feasibility for the resonator working in the nonlinear regions and give the available frequency range and available maximum amplitude of the nonlinear responses.

The paper is organized as follows. In Section 2, the motion equation of the resonator is introduced, and the analytical solution is obtained. The bifurcation characteristics and the jump phenomena are analyzed in Section 3. And in Section 4, we demonstrate the effects of the parameters on the amplitude-frequency curves, the available frequency range and available maximum amplitude. Finally, the conclusions are given in Section 5.

## 2. Periodic Solution of the Comb Drive MEMS Resonator

**Figure 1:**
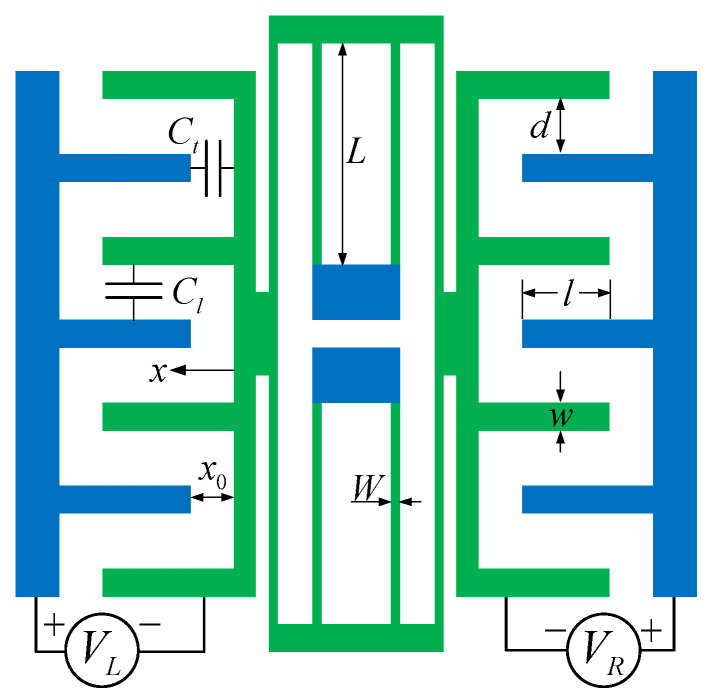
Schematic diagram of comb drive MEMS resonator [5,26].

We consider the comb drive resonator shown in Figure 1. According to refs. [5,26], the governing dynamic equation of the resonator is given as

(1) $$\begin{array}{c} m\ddot{x}+c\dot{x}+k_{L}x+k_{N}x^{3}=\left[\alpha_{2}+\frac{\alpha_{1}}{{\left(x_{0}-x\right)}^{2}}\right]{\left(V_{D}+V_{A}\cos\omega t\right)}^{2}\\ -\left[\alpha_{2}+\frac{\alpha_{1}}{{\left(x_{0}+x\right)}^{2}}\right]{\left(V_{D}-V_{A}\cos\omega t\right)}^{2} \end{array}$$

where

(2) $$\begin{array}{c} K_{L}=\frac{24EI}{L^{3}},K_{N}=\frac{216EI}{35L^{5}},\alpha_{1}=\frac{N\eta A}{2},\\ \alpha_{2}=\frac{\eta (N-1)}{2}\left\{\frac{h}{d}+\frac{1}{\pi }\ln\left[\left[{\left(\frac{w}{d}+1\right)}^{2}-1\right]{\left[\frac{2d}{w}+1\right]}^{\left(1+\frac{w}{d}\right)}\right]\right\} \end{array}$$

where *m* is the proof mass, *N* is the number of fingers on a single side, η is the dielectric constant, *h* is the thickness of the resonator, *w* is the finger width, $x_{0}$ and *l* are the initial lateral separation and overlap between the fixed and moving combs, *d* is the spacing between the fingers, *E* is the Young’s modulus, $I=hW^{3}/12$ is the section modulus, *W* and *L* are the width and length of the supporting beams. $V_{D}$ is the DC voltage, $V_{A}$ is the amplitude of the AC voltage, ω is the driving frequency. Equation (1) can be rewritten as

(3) $$m\ddot{x}+c\dot{x}+k_{L}x+k_{N}x^{3}=4\alpha_{2}V_{D}V_{A}\cos\Omega t+\frac{\alpha_{1}{\left(V_{D}+V_{A}\cos\omega t\right)}^{2}}{{\left(x_{0}-x\right)}^{2}}-\frac{\alpha_{1}{\left(V_{D}-V_{A}\cos\omega t\right)}^{2}}{{\left(x_{0}+x\right)}^{2}}$$

By setting $X=x/x_{0},\tau =\omega_{x}t$, where ${\omega_{x}}^{2}=k_{L}/m$, Equation (3) is written into the non-dimensional form as

(4) $$X^{\prime \prime }+\xi X^{\prime }+X+\gamma X^{3}=E\beta \cos\Omega \tau +E\rho \frac{{\left(1+\beta \cos\Omega \tau \right)}^{2}}{{\left(1-X\right)}^{2}}-E\rho \frac{{\left(1-\beta \cos\Omega \tau \right)}^{2}}{{\left(1+X\right)}^{2}}$$

where

(5) $$\beta =V_{A}/V_{D},\gamma =\frac{k_{N}{x_{0}}^{2}}{m{\omega_{0}}^{2}},\xi =\frac{c}{m\omega_{0}},E=\frac{4\alpha_{2}{V_{D}}^{2}}{m{\omega_{0}}^{2}x_{0}},\rho =\frac{\alpha_{1}}{4\alpha_{2}{x_{0}}^{2}},\Omega =\frac{\omega }{\omega_{x}}$$

In the following the method of averaging [29] is used to solve the periodic response of Equation (4), and the solution is derived as

(6) X=Acosϕ=Acos(Ωτ+θ)

where ϕ=Ωτ, *A* and θ are the amplitude and phase angular of the response. The primary resonance between the driving frequency and the natural frequency of the resonator is considered. By setting $\Omega^{2}=1+\sigma$, where σ is the detuning parameter, the slowly varying equations about the amplitude and phase angular are obtained based on the method of averaging as follows.

(7) $$\begin{array}{c} {A^{\prime }}_{1}=-\frac{1}{2\pi \Omega }\int_{-\pi }^{\pi }f_{1}\sin\phi_{1}d\phi_{1}\\ A_{1}{\theta^{\prime }}_{1}=-\frac{1}{2\pi \Omega }\int_{-\pi }^{\pi }f_{1}\cos\phi_{1}d\phi_{1} \end{array}$$

Equation (7) is solved as

(8) $$\begin{array}{c} A^{\prime }=-\;\frac{E\beta \;\sin\theta +\Omega \;\xi \;A}{2\Omega }+f_{X11}\\ A\theta^{\prime }=-\;\frac{-3\;\gamma \;A^{3}+4\;E\beta \;\cos\theta +4\;\sigma \;A}{8\Omega }+f_{X12} \end{array}$$

where

(9) $$\begin{array}{c} f_{X1}=-\frac{1}{2\pi \Omega }\int_{-\pi }^{\pi }f_{E}\sin\phi d\phi ,\\ f_{X2}=-\frac{1}{2\pi \Omega }\int_{-\pi }^{\pi }f_{E}\cos\phi d\phi ,\\ f_{E}=\frac{\rho E\left(1+\beta \;\cos\Omega \tau \right){\;}^{2}}{{\left(1-X\right)}^{2}}-\frac{\rho E\;{\left(1-\beta \;\cos\Omega \tau \right)}^{2}}{{\left(1+X\right)}^{2}} \end{array}$$

Note that $f_{E}$ in Equation (9) is a fraction of *X*, then the residue theorem [30] is used to solve $f_{X1}$ and $f_{X2}$. By setting z=exp(iϕ), we have

(10) $$\cos\phi =\;\frac{z^{2}+1}{2z},\sin\phi =\frac{z^{2}-1}{2iz}$$

where $i=\sqrt{-1}$. By substituting (10) into $f_{X1}$, and according to the residue theorem, it yields

(11) $$\begin{array}{c} f_{X1}=-\frac{1}{2\pi \Omega }\int_{0}^{2\pi }\frac{\rho E{\left(1+\beta \;\cos\Omega \tau \right)}^{2}}{{\left(1-X\right)}^{2}}-\frac{\rho E\;{\left(1-\beta \;\cos\Omega \tau \right)}^{2}}{{\left(1+X\right)}^{2}}\sin\phi d\phi \\ =-\frac{1}{2\pi \Omega }\oint_{|z|=1}f(z)dz=-\frac{i\varepsilon }{\Omega }\sum \mathrm{Res}[f\left(z\right),z_{k}] \end{array}$$

where

(12) $$f\left(z\right)=\frac{-4\;i\rho E\;\left(z^{2}+1\right)\left(\begin{array}{c} i\beta \;(4\;z^{4}-4\;z^{2}-A^{2}+\;z^{6}A^{2}+\;z^{4}A^{2}-\;z^{2}A^{2})\sin\phi \\ +i\;\beta^{2}\left(A-z^{6}A+z^{2}A-z^{4}A\right)\sin2\;\phi \\ +\beta^{2}\left(\;z^{6}A+z^{4}A+z^{2}A+A\right)\cos2\;\phi \\ -\beta (\;z^{6}A^{2}+4\;z^{4}+3\;z^{4}A^{2}+4\;z^{2}+3\;z^{2}A^{2}+A^{2})\cos\phi \\ +2\;\beta^{2}z^{4}A+2\;\;\beta^{2}z^{2}A+4\;z^{2}A+4\;\;z^{4}A \end{array}\right)}{z{\left(z^{2}A+2\;\;z+A\right)}^{2}{\left(-z^{2}A+2\;z-A\right)}^{2}}$$

$z_{k}$ is the isolated singularity of f(z) contained in the unit circle. There exist three isolated singularities as shown below.

(13) $$z_{1}=\frac{1-\sqrt{1-A^{2}}}{A},z_{2}=-\frac{1-\sqrt{1-A^{2}}}{A},z_{3}=0$$

where $z_{1},z_{2}$ are poles of order 2, and $z_{3}$ is a simple pole, then

(14) $$\mathrm{Res}\left[f\left(z\right),z_{k}\right]=\sum_{k=1,2}\lim_{z\to z_{k}}\frac{d}{dz}\left\{\left(z-z_{k}\right)f\left(z_{k}\right)\right\}+\lim_{z\to 0}zf\left(0\right)$$

Substituting (14) into (11) results in

(15) $$f_{X1}=\frac{4\left(\begin{array}{c} \left[A^{2}\sqrt{1-A^{2}}-2\;\sqrt{1-A^{2}}-2A^{2}+2\;\right]\beta^{2}\sin\theta \cos\theta \\ +[A-A^{3}-4\sqrt{1-A^{2}}]\beta \sin\theta \end{array}\right)}{A^{3}\Omega \;\left(A^{2}-1\right)}$$

Similarly, we have

(16) $$f_{X2}=\frac{\left(\begin{array}{c} \left[8{\left(1-A^{2}\right)}^{2}+2\left(6A^{2}-4-A^{4}\right)\sqrt{1-A^{2}}\;\right]\beta^{2}\cos^{2}\theta \\ +4A[{\left(1-A^{2}\right)}^{2}+\left(A^{2}-1\right)\sqrt{1-A^{2}}]\beta \cos\theta \\ -4\;\beta^{2}{\left(1-A^{2}\right)}^{2}+2\left(A^{4}\beta^{2}-3A^{2}\beta^{2}+A^{4}+2\beta^{2}\right)\sqrt{1-A^{2}} \end{array}\right)}{A^{3}\Omega \;{\left(1-A^{2}\right)}^{2}}$$

By setting the expressions on the right side of Equation (8) to be zero, and eliminating the trigonometric function term of θ (see Appendix A for details), the bifurcation equation for the steady-state periodic response can be obtained as follows.

(17) f(σ,A)=0

**Figure 2:**
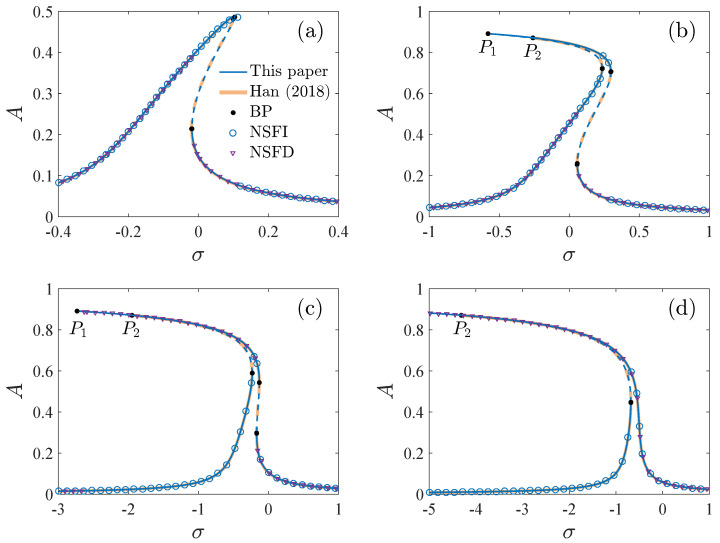
The response curves corresponding to different DC and AC voltages, BP is the bifurcation point, NSFI is the numerical solution as σ is increased, NSFD denotes the numerical solution as σ is decreased, $P_{1}$ and $P_{2}$ are the bifurcation points near the peak of the response curves of this paper and ref. [26] by Han et al. (2018), respectively. (**a**) $V_{D}=10\;V,\;V_{A}=10\;\mathrm{mV}$. (**b**) $V_{D}=10\;V.\;V_{A}=18\;\mathrm{mV}$. (**c**) $V_{D}=15\;V,\;V_{A}=12\;\mathrm{mV}$. (**d**) $V_{D}=20\;V,\;V_{A}=9\;\mathrm{mV}$.

The stability of the periodic solution is determined by the eigenvalues of the Jacobian matrix of Equation (8) [26]. Figure 2 shows the response curves given by Equation (17) corresponding to different DC and AC voltages. The values of the parameters are selected as Table 1 in the following calculations unless explicitly stated. We also present the results of ref. [26] and the numerical results of Equation (4) obtained by the fourth-order Runge-Kutta method. To illustrate the results clearly, we give two points $P_{1}$ and $P_{2}$ in Figure 2b–d, which are the bifurcation points near the peak of the solution curves of this paper and ref. [26], respectively. When the amplitude is small (A≤0.8), the solution developed in this paper is similar to that of ref. [26], and both of them are in good agreement with the numerical solution. As the amplitude is large (A>0.8), the solution of this paper is quite different from that of ref. [26]. $P_{1}$ is on the right side of $P_{2}$ in Figure 2b,c. And $P_{1}$ does not exist in Figure 2d. The solution of this paper seems to be closer to the numerical solution. Further, corresponding to different parameters, the topological structure of the response curves may be different, which may lead to different dynamic behaviors. Thus, it is necessary to analyze the bifurcation characteristics of Equation (17).

## 3. Singularity Analysis on the Periodic Response of the Resonator

In this section, the singularity theory [31,32] is used to study the influence of parameters on the bifurcation behaviors of the resonator. The transition sets of Equation (17) are defined as D=B∪H∪DL, where B, H, DL denote the bifurcation set, the hysteresis set and the double limit point set. The expressions of the transition sets are given in Table 2.

**Figure 3:**
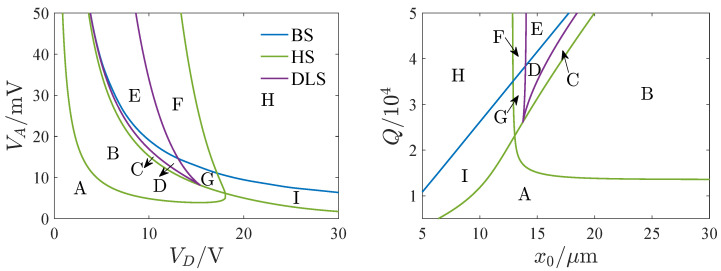
Transition sets on $V_{D}-V_{A}$ and $x_{0}-Q$ planes, BS, HS, and DLS denote bifurcation set, hysteresis set, and double limit point set.

**Figure 4:**
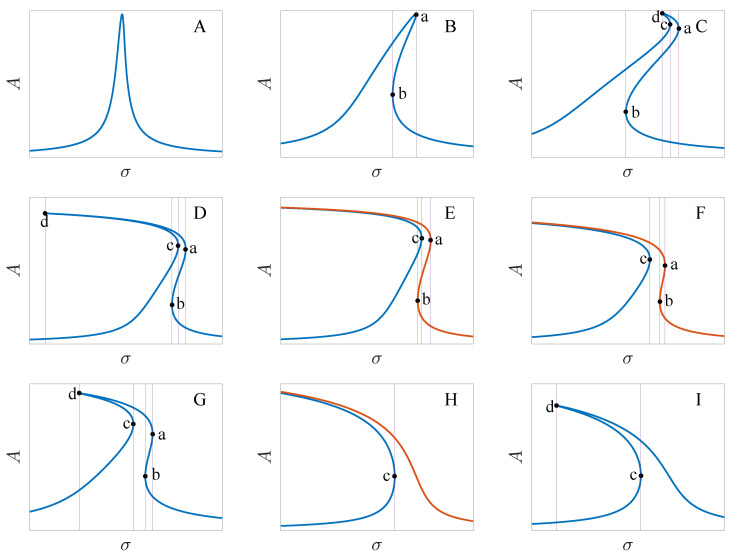
Bifurcation diagrams corresponding to different persist regions of Figure 3, where Figures (**A**) to (**I**) correspond to regions A through I, respectively.

Figure 3 shows the transition sets on the $V_{D}-V_{A}$ and $x_{0}-Q$ plane. The transition sets divide the parameter planes into 9 persistent regions. Figure 4 gives the bifurcation diagram corresponding to different parameter regions. To explain the topological structure of the bifurcation curves clearly, we define four key points as a through d which are the turning points of the bifurcation curves. Note that the bifurcation diagrams have a single solution branch in regions A, B, C, D, G and I. In region A, the turning point does not appear, and the bifurcation curve has a single solution with any value of σ. In regions B and I, the bifurcation curves have two turning points, and there exist three solutions between the two key points. In regions C, D and G, four key points all appear. In region C, point d is on the right side of b. In region D, point d is on the left side of b, which leads to the increase of the multisolution region. In region G, point c is on the left side of b. There exist two multisolution regions, and the single solution region lies between points b and c. In regions E, F and H, the bifurcation diagrams have two solution branches. Only one turning point exists in region H. Point c is on the right side of b point in region E, and on the left side of b in region F.

Further, we note the bifurcation curves are hardening in region B and softening in regions H and I. In regions C through G, the bifurcation curves are mixtures of hardening and softening.

**Figure 5:**
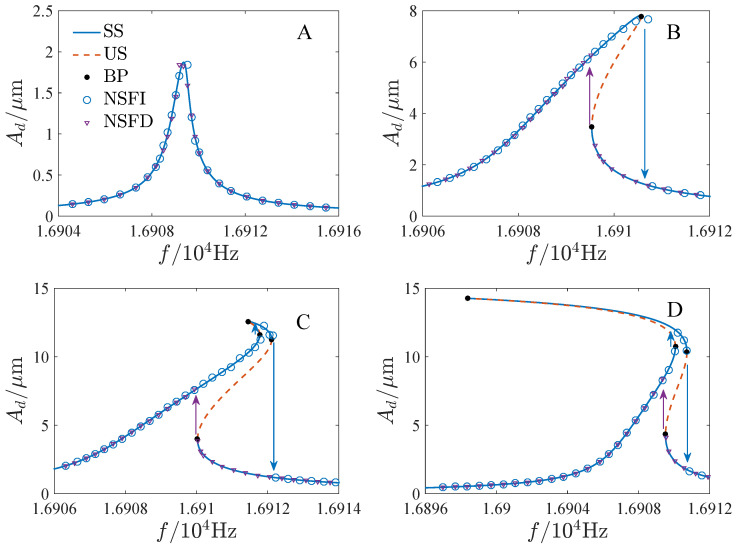

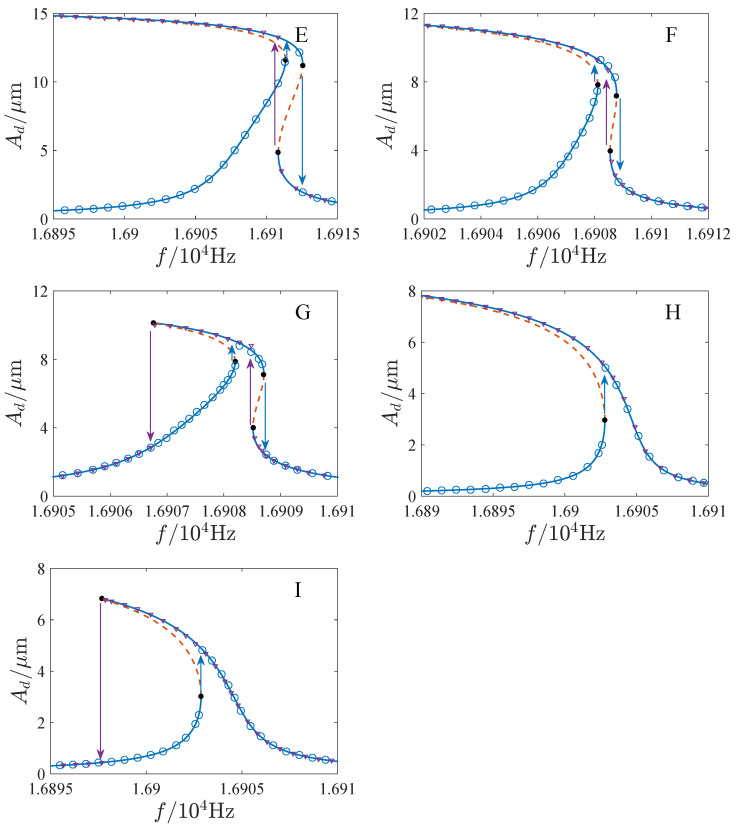
Amplitude-frequency curves corresponding to different regions of Figure 3, where Figures (**A**) to (**I**) correspond to regions A through I, respectively. SS is stable analytical solution, US is unstable analytical solution, BP is bifurcation point, NSFI denotes numerical solution as driving frequency is increased, and NSFD denotes numerical solution as driving frequency is decreased.

To illustrate the dynamic behaviors of the previous bifurcation diagrams, Figure 5 gives the amplitude-frequency curves represented by the physical parameters. $A_{d}=Ax_{0}$ is the dimensional amplitude and *f* is the driving frequency. The corresponding parameter values are given in Table 3. Compared with that of the existing investigations, we show more abundant jump phenomena. Jump does not happen in region A. In regions B and I, the response respectively jumps once as the frequency is increased and decreased. Jump phenomena happen twice in regions C and D with the increase of the driving frequency. In region G, the jump will occur twice as the frequency is increased and decreased. In regions E, F and H, the response will not jump down when the frequency is decreased. Figure 5 also presents the numerical results, which meet the analytical results very well.

## 4. Influence of the Parameters on the Response of the Resonator

**Figure 6:**
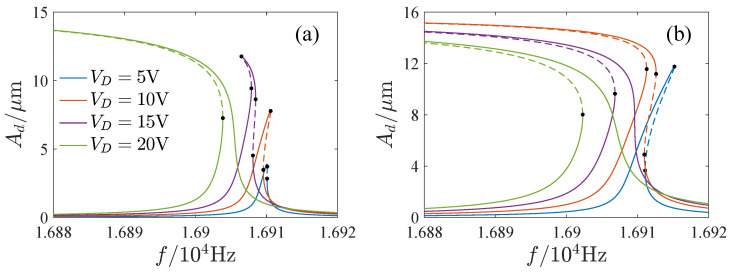
Effects of DC voltage on amplitude-frequency curves. (**a**) $V_{A}=10\;\mathrm{mV}$. (**b**) $V_{A}=30\;\mathrm{mV}$.

**Figure 7:**
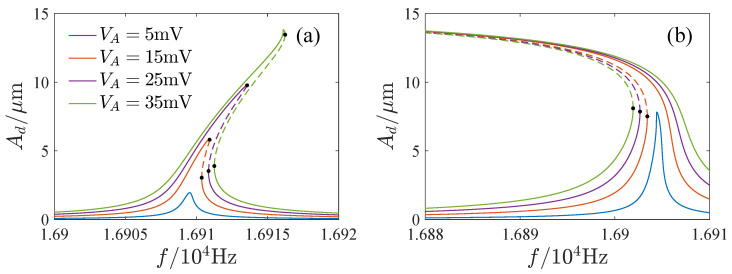
Effects of AC voltage on amplitude-frequency curves. (**a**) $V_{D}=5\;V$. (**b**) $V_{D}=20\;V$.

**Figure 8:**
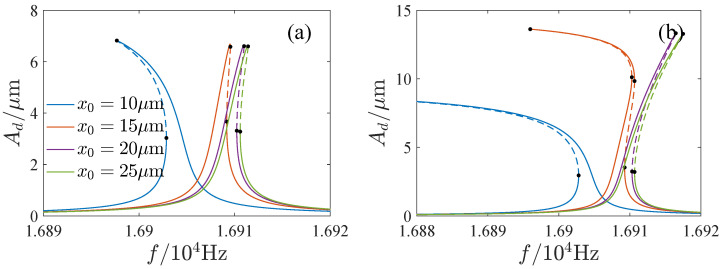
Effects of lateral separation on amplitude-frequency curves. (**a**) Q=20,000. (**b**) Q=40,000.

**Figure 9:**
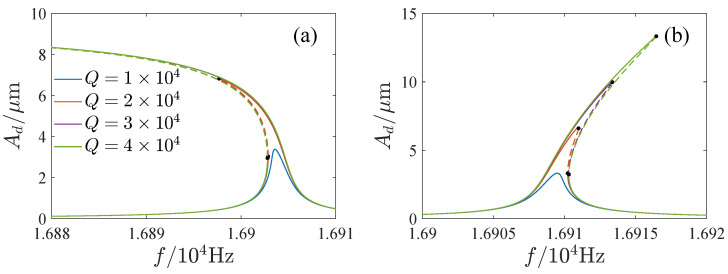
Effects of quality factor on mplitude-frequency curves. (**a**) $x_{0}=10\;\mu m$. (**b**) $x_{0}=20\;\mu m$.

The influence of the parameters is important for the design and operation of resonators. Figure 6, Figure 7, Figure 8 and Figure 9 show the effects of the DC voltage, AC voltage, lateral separation and quality factor on the amplitude-frequency curves. With the increase of the DC voltage, the amplitude-frequency curves change from hardening to softening. Before the response turns into softening, the largest amplitude of the response increases with the DC voltage. As the response becomes softening, the largest amplitude decreases with the DC voltage. The response changes from softening to hardening with the increase of the lateral separation. As the response is softening, the amplitude increases with the separation. When the response ceases to be softening, the separation has little effect on the amplitude. The response increases along the backbone curves with the AC voltage and quality factor.

**Figure 10:**
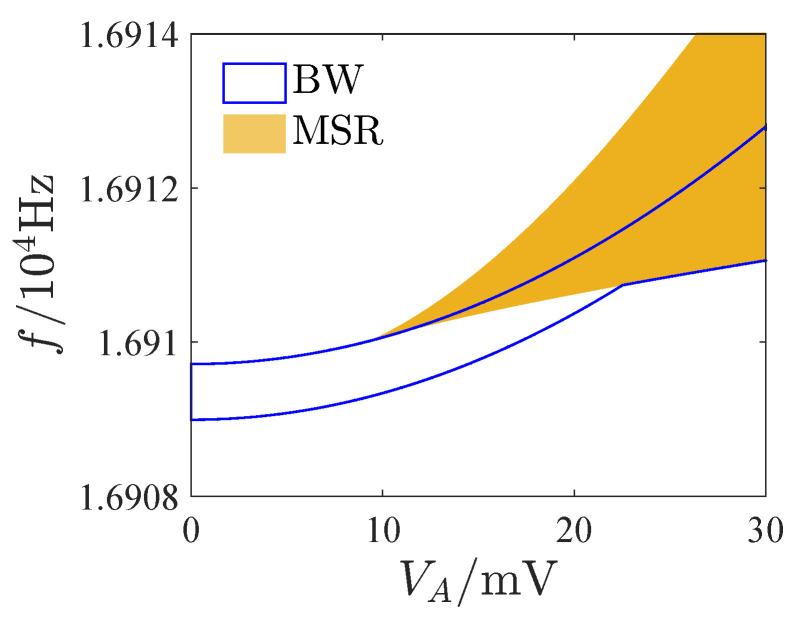
Influence of AC voltage on 3 dB bandwidth of resonator. BW is bandwidth; MSR denotes multisolution region.

**Figure 11:**
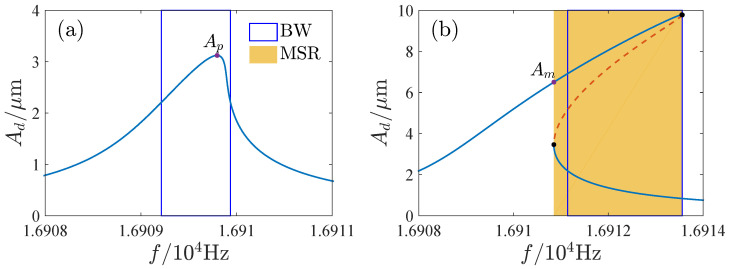
Amplitude-frequency curves with different AC voltages. BW is bandwidth; MSR denotes multisolution region. (**a**) $V_{A}=8\;\mathrm{mV}$. (**b**) $V_{A}=25\;\mathrm{mV}$.

Bandwidth is very important for resonators. We studied the influence of the AC voltage on the 3 dB bandwidth of the resonator, as shown in Figure 10. It shows with the increase of the AC voltage, a multisolution phenomenon appears in the response. The multisolution region gradually erodes the bandwidth as the AC voltage is further increased. When the AC voltage is large, the bandwidth is wholly included in the multisolution region. The resonator cannot work stably in the multisolution region without a phase-locked loop, since the response may jump to other solution as the system is disturbed, which may result in incorrect output. Figure 11 presents the amplitude-frequency curves with different AC voltages. Figure 11a,b are the typical linear and nonlinear responses. Although the bandwidth of the response curve is completely included in the multisolution region in Figure 11b, the largest amplitude of the single solution part is greater than the peak amplitude of Figure 11a. The resonator may have a good response in the single solution region of Figure 11b. Hence, it is of great significance to study the available frequency range and maximum amplitude of the single solution region of the response curves of the open-loop operated MEMS resonator. For this reason, we define AFR (the available frequency range of the resonator) as a continuous 3 dB frequency range of the maximum amplitude in the single solution region of the response curve (excluding the multisolution regions).

**Figure 12:**
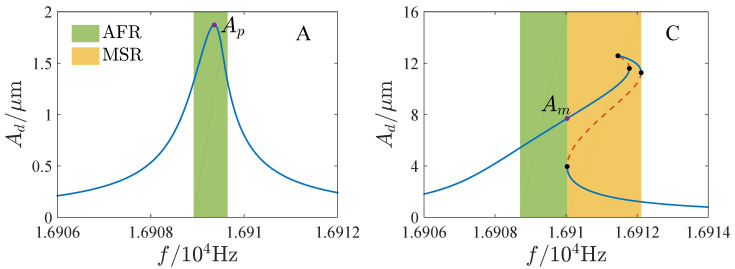

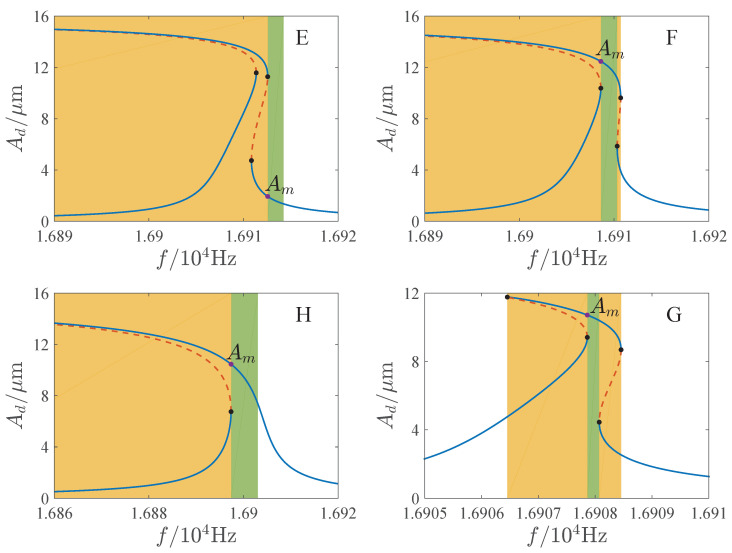
Available frequency range and available maximum amplitude of corresponding parameter regions A, C, E, F, G and H. AFR = available frequency range; MSR = multisolution region.

**Figure 13:**
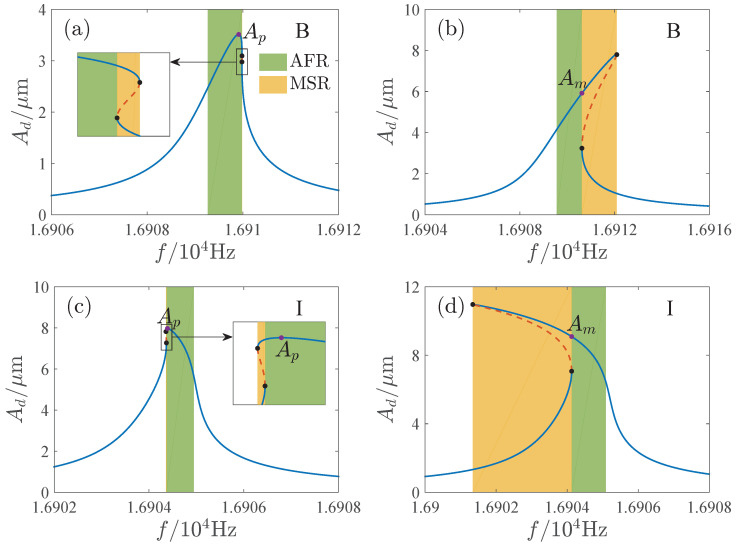

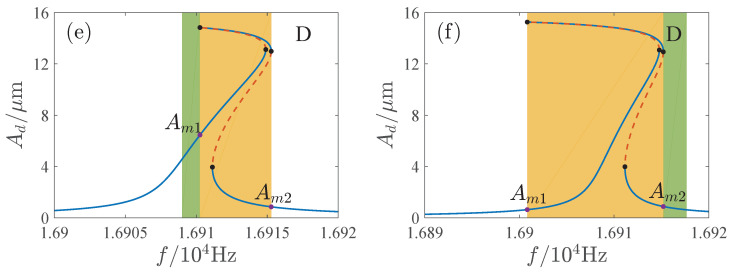
Available frequency range and available maximum amplitude of corresponding parameter regions B, I, and D, where (**a**,**b**) correspond to region B, (**c**,**d**) correspond to region I, (**e**,**f**) correspond to region D. AFR = available frequency range; MSR = multisolution region.

Figure 12 and Figure 13 show the AFR and maximum amplitudes for different parameter regions. The AFR is also its bandwidth in region A. In region C, the AFR is on the left side of the multisolution region. The AFR of E and H is on the right side of the multisolution region. And the AFR of F and G is between the two multisolution regions. In regions B and I, there are two possibilities. As the peak of the amplitude-frequency curve lies in the single solution region, AFR is determined by the peak amplitude $A_{p}$ as shown in Figure 13a,c. When the peak of the amplitude-frequency curve is included in the multisolution region, AFR is determined by the maximum amplitude of the single solution part $A_{m}$ as shown in Figure 13b,d. In region D, the values of the maximum amplitudes of the left and right single solution regions should be considered. When $A_{m1}$ is greater than $A_{m2}$ (see Figure 13e), AFR is on the left side of the multisolution region, and in contrast (see Figure 13f), it is on the right side.

**Figure 14:**
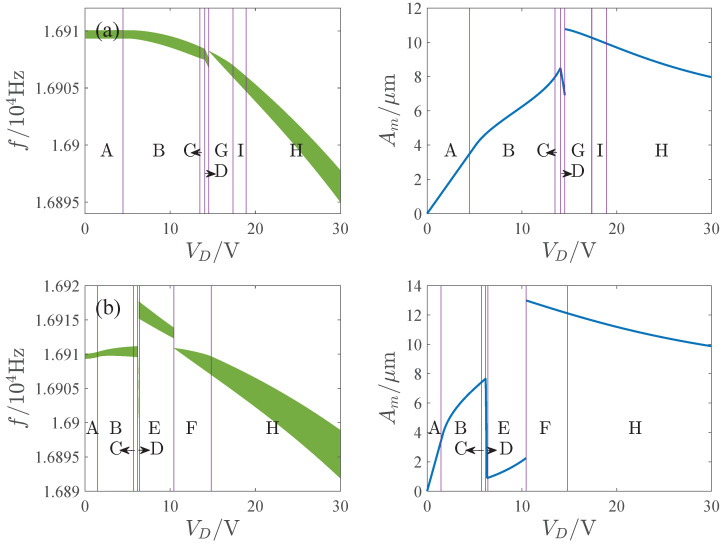
Influence of DC voltage on APR and available maximum amplitude. (**a**) $V_{A}=10\;\mathrm{mV}$. (**b**) $V_{A}=30\;\mathrm{mV}$.

**Figure 15:**
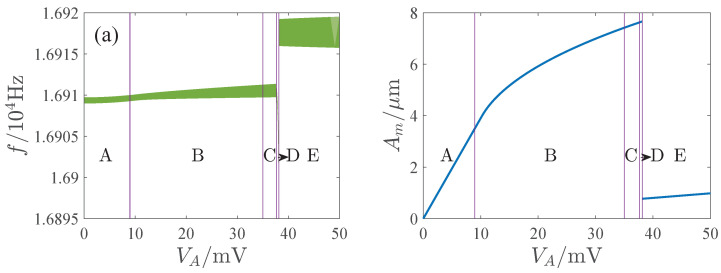

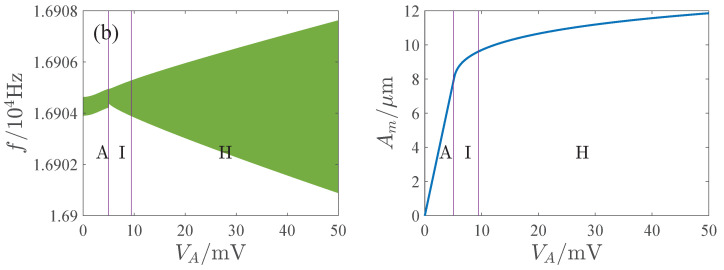
Influence of AC voltage on APR and available maximum amplitude. (**a**) $V_{D}=5\;V$. (**b**) $V_{D}=20\;V$.

**Figure 16:**
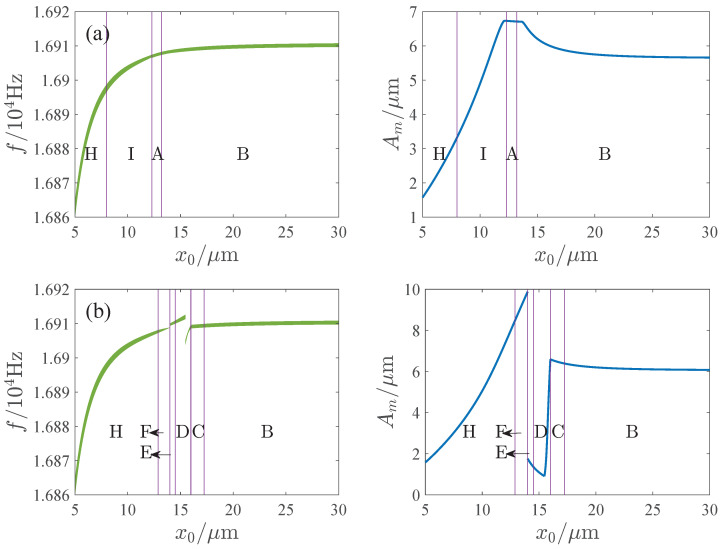
Influence of lateral separation on APR and available maximum amplitude. (**a**) Q=20,000. (**b**) Q=40,000.

**Figure 17:**
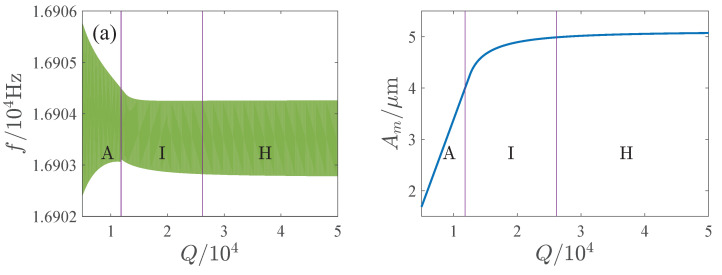

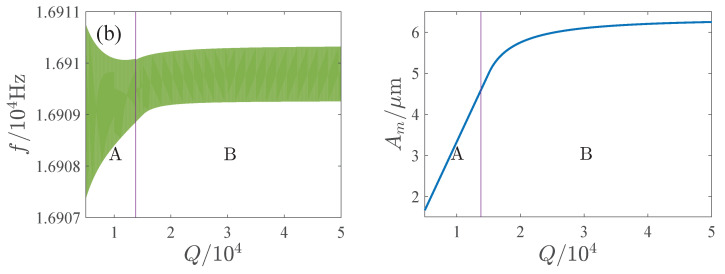
Influence of quality factor on APR and available maximum amplitude. (**a**) $x_{0}=10\;\mu m$. (**b**) $x_{0}=20\;\mu m$.

Figure 14, Figure 15, Figure 16 and Figure 17 show the effects of the DC voltage, AC voltage, lateral separation and quality factor on the AFR and available maximum amplitude. The green region is the AFR and $A_{m}$ denotes the maximum available amplitude. In the parameter region E, $A_{m}$ is very small. And $A_{m}$ may change dramatically in region D. Corresponding to the parameter regions F and G, although $A_{m}$ is large, the AFR is sensitive to the parameters. For some parameters, the AFR is too small for the operation of the resonator. Thus, the parameter regions D through G are unsuitable for the resonator. Comparatively speaking, the response curves of softening or hardening characteristics, such as those of regions B, H, and I, have enough AFR and large $A_{m}$, which may be more appropriate for the operation of the MEMS resonators than those of the mixture characteristic.

We also find that in the softening characteristic regions H and I, $A_{m}$ decreases with the DC voltage and increases with the AC voltage, lateral separation and quality factor. In the hardening characteristic region B and the linear response region A, $A_{m}$ increases with the DC voltage, AC voltage and quality factor, decreases with the lateral separation, and gradually tends to a constant with the increase of the lateral separation.

## 5. Conclusions

We investigate the bifurcation characteristics of a comb drive MEMS resonator in this paper. The dynamic equation with fractional nonlinearity is solved to give a more accurate analytical solution of the periodic response by the method of averaging and the residue theorem. The singularity theory is used to get the transition sets on the DC-AC voltage plane and the lateral separation-quality factor plane, which divide the planes into nine persistent regions. The topological structures of the bifurcation diagrams corresponding to different parameter regions are analyzed. Abundant jump phenomena of the periodic responses varied with the driving frequency are present.

The influences of the parameters on the amplitude-frequency response are investigated. The results demonstrate that the amplitude-frequency curves change from hardening to softening with the increase of the DC voltage. The effects of the lateral separation are the opposite. The amplitude-frequency curves increase along the backbone curves with the AC voltage and quality factor.

The feasibility of the open-loop operated resonator working in the nonlinear regions is further discussed. The available frequency range and the available maximum amplitude of the nonlinear responses are present. We find that the response curves of softening or hardening characteristics have enough AFR and large available amplitude, which may be more appropriate for the operation of the MEMS resonators than those of the mixture characteristics.

## Figures and Tables

**Table 1:** Parameters of comb drive MEMS resonator [5].

Parameters	Values
Proof mass, *m*	$5.73\times 10^{-11}$ kg
Length of the supporting beam, *L*	185.3 μm
Width of the supporting beam, *W*	1.9 μm
Structure thickness, *h*	2 μm
Young’s modulus, *E*	150 GPa
Finger width, *w*	2 μm
Initial overlap, *l*	20 μm
Finger spacing, *d*	2 μm
Initial lateral separation, $x_{0}$	16 μm
Dielectric constant, η	$8.85\times 10^{-12}$ F/m
Number of fingers, *N*	60
DC voltage, $V_{D}$	10 V
Amplitude of the AC voltage, $V_{A}$	10 mV
Quality factor	23,400

**Table 2:** Expressions of transition sets of singularity theory [31,32].

Transition Sets	Expressions
Bifurcation set B	$f=f_{\sigma }=f_{A}=0$
Hysteresis set H	$f=f_{A}=f_{AA}=0$
Double limit point set DL	$f\left(A_{\left(i\right)},\sigma \right)=f_{A_{\left(i\right)}}\left(A_{\left(i\right)},\sigma \right)=0,i=1,2$ and $A_{\left(1\right)}\neq A_{\left(2\right)}$

**Table 3:** Values of parameters corresponding to Figur Figure 5A–I.

Figures	$V_{D}$/V	$V_{A}$/mV	$x_{0}/\mu$m	*Q*
Figure 5A	6	4	16	23,400
Figure 5B	10	10	16	23,400
Figure 5C	10	16	16	23,400
Figure 5D	12	15	16	23,400
Figure 5E	10	28	16	23,400
Figure 5F	10	10	13.5	46,000
Figure 5G	10	10	13.5	30,000
Figure 5H	10	10	10	40,000
Figure 5I	10	10	10	20,000

## Appendix A

It can be obtained from the first equation of Equation (8) that

(A1) $$\sin\theta =\frac{C_{3}}{C_{1}+C_{2}\cos\theta }$$

where

(A2) $$\begin{array}{c} C_{1}=E\beta \left[\;\left(A^{2}-1\right)\left(A^{3}+8\;A\rho \right)+8\;A\rho \sqrt{1-A^{2}}\right]\\ C_{2}=8E\beta {\;}^{2}\rho \left[\left(2-A^{2}\right)\sqrt{1-A^{2}}+2\;A^{2}-2\right]\\ C_{3}=-\xi \;A^{4}\Omega \;\left(A^{2}-1\right) \end{array}$$

Accordingly we have

(A3) $$\cos^{2}\theta +\sin^{2}\theta -1=\cos^{2}\theta +\frac{{C_{3}}^{2}}{{\left(C_{1}+C_{2}\cos\theta \right)}^{2}}-1=0$$

The molecules of (A3) can be obtained as

(A4) $$f_{1}=\left(1-\cos^{2}\theta \right){\left(C_{1}+C_{2}\cos\theta \right)}^{2}-{C_{3}}^{2}=0$$

The second equation of Equation (8) is rewritten in the following form.

(A5) $$B_{1}\cos^{2}\theta +B_{2}\cos\theta +B_{3}=0$$

It can be solved from (A5) as

(A6) $$\cos^{2}\theta =-\frac{B_{2}\cos\theta +B_{3}}{B_{1}}$$

where

(A7) $$\begin{array}{c} B_{1}=-16\;E\beta^{2}\rho \left[\left(A^{4}-6\;A^{2}+4\;\right)\sqrt{1-A^{2}}-4\;{\left(1-A^{2}\right)}^{2}\right]\;\\ B_{2}=-4\;AE\beta \;\left[\left(A^{2}-8\;\rho \right){\left(1-A^{2}\right)}^{2}+\left(8\;-16\;A^{2}\right)\rho \sqrt{1-A^{2}}\right]\\ B_{3}=\left(3\gamma A^{6}-4\sigma A^{4}+32E\beta^{2}\rho \right){\left(1-A^{2}\right)}^{2}+16E\rho \left(A^{4}\beta^{2}-3A^{2}\beta^{2}+2\beta^{2}+A^{4}\right)\sqrt{1-A^{2}} \end{array}$$

By substituting (A6) into (A4), the power of cosθ in (A4) can be reduced until the expression of cosθ is obtained. Substituting it into Equation (A5) can find the expression of the bifurcation equation.

## References

[1] Cao Y., Wang P., Li J., Xie H. (2021). Temperature stability study of resonant angular scanning micromirrors with electrostatic comb-drive actuators. Sens. Actuators A-Phys..

[2] Sheikhaleh A., Jafari K., Abedi K. (2019). Design and analysis of a novel MOEMS gyroscope using an electrostatic comb-drive actuator and an optical sensing system. IEEE Sens. J..

[3] Kavitha S., Joseph Daniel R., Sumangala K. (2016). Design and analysis of MEMS comb drive capacitive accelerometer for SHM and seismic applications. Measurement.

[4] Ghasemi S., Afrang S., Rezazadeh G., Darbasi S., Sotoudeh B. (2020). On the mechanical behavior of a wide tunable capacitive MEMS resonator for low frequency energy harvesting applications. Microsyst. Technol..

[5] Elshurafa A.M., Khirallah K., Tawfik H.H., Emira A., Abdel Aziz A.K.S., Sedky S.M. (2011). Nonlinear dynamics of spring softening and hardening in folded-mems comb drive resonators. J. Microelectromech. Syst..

[6] Truong B.D., Le C.P., Halvorsen E. (2019). On the lateral instability analysis of mems comb-drive electrostatic transducers. Sensors.

[7] Tusset A.M., Balthazar J.M., Bassinello D.G., Pontes B.R., Felix J.L.P. (2012). Statements on chaos control designs, including a fractional order dynamical system, applied to a “MEMS” comb-drive actuator. Nonlinear Dyn..

[8] Chang W., Zorman C. (2008). Electrical characterization of microelectromechanical silicon carbide resonators. Sensors.

[9] Ramanan A., Teoh Y., Ma W., Ye W. (2016). Characterization of a laterally oscillating microresonator operating in the nonlinear region. Micromachines.

[10] Guo D., Zhu Y. (2010). The effects of structure defects on the performance of a micro comb resonator. Math. Probl. Eng..

[11] Mukherjee B., Swamy K.B.M., Sen S. (2016). Dynamic characteristics of voltage induced reciprocated bending in double cantilever configuration of asymmetric comb drive MEMS. Microsyst. Technol..

[12] Sheikhaleh A., Jafari K., Abedi K. (2015). Dynamically balanced folded-beam suspensions for resonators. J. Microelectromech. Syst..

[13] Chen D., Wang Y., Guan Y., Chen X., Liu X., Xie J. (2018). Methods for nonlinearities reduction in micromechanical beams resonators. J. Microelectromech. Syst..

[14] Khirallah K. (2013). Parametric excitation, amplification, and tuning of MEMS folded-beam comb drive oscillator. J. Microelectromech. Syst..

[15] Taherian S., Ganji B.A., Jafari-Talookolaei R. (2020). A novel MEMS tunable comb resonator with non-uniform varied finger lengths. IEEE Sens. J..

[16] Zhang Q., Ren H., Han J. (2016). Nonlinear dynamics of folded-MEMS comb drive resonators with time-delayed control. J. Shock Vib..

[17] Nashat S.E.D., AbdelRassoul R., Abd El Bary A.E.M. (2018). Design and simulation of RF MEMS comb drive with ultra-low pull-in voltage and maximum displacement. Microsyst. Technol..

[18] Kozinsky I., Postma H.W.C., Bargatin I., Roukes M.L. (2006). Tuning nonlinearity, dynamic range, and frequency of nanomechanical resonators. Appl. Phys. Lett..

[19] Nichol J.M., Hemesath E.R., Lauhon L.J., Budakian R. (2009). Controlling the nonlinearity of silicon nanowire resonators using active feedback. Appl. Phys. Lett..

[20] Zhang Y., Yoshioka Y., Iimori M., Qiu B., Liu X., Hirakawa K. (2021). Thermal tuning of mechanical nonlinearity in GaAs doubly-clamped MEMS beam resonators. Appl. Phys. Lett..

[21] Huang L., Soskin S.M., Khovanov I.A., Mannella R., Ninios K., Chan H.B. (2019). Frequency stabilization and noise-induced spectral narrowing in resonators with zero dispersion. Nat. Commun..

[22] Zhang W., Meng G. (2007). Nonlinear dynamic analysis of electrostatically actuated resonant mems sensors under parametric excitation. IEEE Sens. J..

[23] Kacem N., Hentz S. (2009). Bifurcation topology tuning of a mixed behavior in nonlinear micromechanical resonators. Appl. Phys. Lett..

[24] Khan Y., Akbarzade M. (2012). Dynamic analysis of nonlinear oscillator equation arising in double-sided driven clamped microbeam-based electromechanical resonator. Z. Naturforsch. A.

[25] Khan Y., Al-Hayani W. (2013). A Nonlinear model arising in the buckling analysis and its new analytic approximate solution. Z. Naturforsch. A.

[26] Han J., Li L., Jin G., Feng J., Li B., Jia H., Ma W. (2018). Vibration identification of folded-MEMS comb drive resonators. Micromachines.

[27] Zhong Z., Zhang W., Meng G., Wu J. (2013). Inclination effects on the frequency tuning of comb-driven resonators. J. Microelectromech. Syst..

[28] Ma H., Zhang Q., Chen T., Li L. (2018). Static and dynamic characteristics of MEMS comb resonators considering fringe effect. J. Shock Vib..

[29] Nayfeh A.H., Mook D.T. (1995). Nonlinear Oscillations.

[30] Brown J.W., Churchill R.V. (2009). Complex Variables and Applications.

[31] Golubisky M., Schaeffer D.G. (1985). Singularities and Groups in Bifurcation Theory.

[32] Hou L., Su X., Chen Y. (2019). Bifurcation modes of periodic solution in a duffing system under constant force as well as harmonic excitation. Int. J. Bifurc. Chaos.

